# Special Issue: Bioceramics, Bioglasses, and Gels for Tissue Engineering

**DOI:** 10.3390/gels9070586

**Published:** 2023-07-21

**Authors:** Arish Dasan, Ashokraja Chandrasekar

**Affiliations:** FunGlass, Alexander Dubček University of Trenčín, 911 50 Trenčín, Slovakia; ashokraja.chandrasekar@tnuni.sk

Undoubtedly, biomaterials such as bioceramics, bioactive glasses, and gels have attracted a wide range of research interest in the field of tissue engineering (TE), as they facilitate the essential support and environment for cells to grow, differentiate, and, specifically, regenerate new tissues [1,2,3,4,5]. Orthopaedic and dental implants are increasingly being used to treat patients of all ages who are missing bones or teeth. Biocompatibility, mechanical stability, corrosion resistance, and antimicrobial resistance are just some of the criteria that must be met for a material to be used as a biomaterial or an implant [6,7]. Although biomaterials offer many benefits, challenges still exist, for instance, achieving the desirable mechanical properties, controlling their degradation properties, and ensuring their long-term stability. From this perspective, many researchers working on advancements in biomaterials by adopting novel processing and manufacturing techniques such as 3D printing continue to address these challenges and expand the possibilities for their applications in TE [8,9,10]. Bioceramics, such as hydroxyapatite (HA) and tricalcium phosphate (TCP), are commonly used in bone TE applications [11,12]. High-strength and aesthetic bioceramics such as zirconia find extensive use in dental applications [13,14]. Bioceramics are also used as a coating on commercial metallic implants, thereby enhancing their biocompatibility, bone integration, and improving the lifetime of the implant [15,16]. Apart from hard tissue, recently, bioceramics have been explored in soft tissue engineering applications, for instance, skin regeneration, the regeneration of periodontal tissues, the cure of articular cartilage, myocardial necrosis, and neovascularization growth [1,2,3,17]. Further investigations, by modifying their composition and surface properties, for example, are likely to lead to the potentiality of regenerating soft tissues.

One of the biomaterials that has transformed contemporary biomaterial-driven regenerative medicine is bioactive glass, which has created novel applications in biomedicine such as soft tissue repair and drug delivery [6]. Bioglass^®^, pioneered by Hench, is just one example of how much progress has been made in biomaterials in the last few decades [18]. It would be too modest to acknowledge that, among man-made materials, it is a marvel for its ability to both chemically bond to the host bone and promote cell proliferation. Bioactive glasses’ main benefit is the controlled release of therapeutic ions from their silica matrix, which stimulates protein and cellular attachment and aids in the repair of damaged bones by the means of cell proliferation [19,20,21]. Many varieties of bioactive glasses, including the typical 45S5 silicate glass (45S5 or Bioglass^®^), antibacterial bioactive glasses (S53P4 or BonAlive^®^), and borate-based glasses (13-93B3 bioactive glass), have been produced and marketed [22]. Recent advances in the development of bioactive glasses for bone regeneration have relied on porous scaffolds that can serve as 3D representations of bone structures. In addition to conventional foaming procedures and template-based methods, a variety of additive manufacturing techniques are currently being employed to construct scaffolds from melt and sol-gel-derived glasses. These techniques permit greater control over the pore structure and formation of the scaffolds [22].

Gels are widely used in TE because of their unique properties, such as high water content, softness, and the provision of a three-dimensional network environment, which make them suitable for cell growth, nutrient diffusion, and tissue regeneration [23,24,25]. Hydrogels formed from natural components such as collagen, fibrin, and gelatin are the most used in TE due to their ability to mimic the native extracellular matrix (ECM). Hydrogels can serve as delivery vehicles for potential therapeutic agents, such as drugs and growth factors, enabling their localized and sustained release [25]. In addition, the inclusion of potential therapeutic inorganic biomaterials within a gel network can stimulate by delivering the physical and biochemical cues necessary for tissue development [25]. Considering their limitations, the lack of mechanical strength that restricts their use in load-bearing applications, achieving a detailed microstructure, and controlling the degradation rate are the main challenges. However, it should be noted that the plusses and constraints can vary depending on the gel materials, formulations, and applications. Research teams are continually working to improve reliable gel systems and minimize their limitations.

In recent years, significant research attention has been paid to utilizing biomaterials as feedstock in AM technology [26,27,28]. The advent of such technology has enabled new possibilities for producing high-performance components directly from customized digital models, which is not feasible using conventional fabrication techniques. It is also possible to manufacture optimal pore sizes with interconnection. Recent emerging approaches, such as omics-based approaches, can allow for a comprehensive study of the regenerative potential of biomaterials [1]. Another hot topic is to develop engineered biomaterials that are (i) bio-instructive (designed to provide chemical and physical cues to guide cellular behavior), (ii) biomimetic (aimed at replicating the structure, including the microstructure and properties, including the chemical and physical ones of natural tissues or organs), and (iii) bioresponsive (designed to exhibit specific responses when exposed to biological stimuli or signals).

Advancements in interdisciplinary fields such as chemistry, physics, material science, nanotechnology, manufacturing technology, and bioengineering have led to the development of a wide range of biomaterial components. These materials are being increasingly explored for a wide range of applications, including regenerative medicine, biosensors, bioelectronics, and personalized implants, with the aim of improving human health and well-being.

This Special Issue collection of articles represents the keen and diverse research ensuing toward innovative functionalities and technologies in the biomedical sector. Atkinson I et al. [29] fabricated composite scaffolds consisting of poly methyl methacrylate (PMMA) Cerium-doped mesoporous bioactive glass (MBG), by means of the phase separation method. In addition, they studied the effect of ceria addition and thereby the property changes, particularly the crystallization behavior, of the SiO_2_–CaO–P_2_O_5_–CeO_2_ system [30]. Yergeshov AA et al. [31] investigated the in vivo and in vitro effects of metal ion (Cu, Co, and Zn)-doped biodegradable macroporous cryogels. The research performed by Dascalu LM et al. [32] revealed the adjuvant effect of natural photosensitizers, based on curcuma extract and oregano essential oil, on induced periodontal diseases. Mosas KKA et al. [15] apprized the recent developments in biomaterials and coatings for different biomedical implants. Another review article [33] discussed soft tissue repair using nanoparticle-modified biocompatible polymers including hydrogels. Schrade S et al. [34] found that gelatin nanoparticles can be used for targeted dual drug release out of aleginate-di-aldehyde-gelatin gels. Calcium magnesium silicate (CaO-MgO-SiO_2_)-based bioceramics, containing bioactive phases of diopside, akermanite, and merwinite, were prepared by Alecu AE et al. [35] using the sol-gel method followed by thermal treatment. Reyes-Peces MV and their research group [36] fabricated bioactive 3D scaffolds based on silica, gelatin, and β-tricalcium phosphate by the means of robocasting additive manufacturing technology and laser micromachining. Phetcharat P et al. [37] studied the influence of the addition of copper iodide nanoparticles on poly(vinyl alcohol) (PVA) liquid bandages for wound healing applications. Another interesting review article by Kishani M and their team [38] summarized the recent advancements in scaffolds, particularly those fabricated from bio-based natural materials. Miyamoto Y [39] extensively reviewed cell sheets, vitrified hydrogel membranes, and their cryopreservation applications in regenerative and cellular medicine.
